# Early results of an integrated maternal, newborn, and child health program, Northern Nigeria, 2009 to 2011

**DOI:** 10.1186/1471-2458-13-1034

**Published:** 2013-10-31

**Authors:** Sally E Findley, Omolara T Uwemedimo, Henry V Doctor, Cathy Green, Fatima Adamu, Godwin Y Afenyadu

**Affiliations:** 1 Department of Population and Family Health, Mailman School of Public Health, Columbia University, 60 Haven Avenue, New York, NY 10032, USA; 2 Pediatric Global Health Program, Cohen Children’s Medical Centre of New York, Division of General Pediatrics, New Hyde Park, New York, USA; 3 PRRINN-MNCH Programme, No. 2 Mallam Bakatsine Street, Nassarawa GRA, Kano, Nigeria; 4 Health Partners International, Waterside Centre, North Street, Lewes, East Sussex BN7 2PE, United Kingdom

**Keywords:** Infant mortality, Newborn and child health, Nigeria

## Abstract

**Background:**

This paper describes early results of an integrated maternal, newborn, and child health (MNCH) program in Northern Nigeria where child mortality rates are two to three times higher than in the southern states. The intervention model integrated critical health systems changes needed to reinvigorate MNCH health services, together with community-based activities aimed at mobilizing and enabling women to make changes in their MNCH practices. Control Local Government Areas received less-intense statewide policy changes.

**Methods:**

The impact of the intervention was assessed using a quasi-experimental design, comparing MNCH behaviors and outcomes in the intervention and control areas, before and after implementation of the systems and community activities. Stratified random household surveys were conducted at baseline in 2009 (n = 2,129) and in 2011 at follow-up (n = 2310), with women with births in the five years prior to household surveys. Chi-square and t-tests were used to document presence of significant improvements in several MNCH outcomes.

**Results:**

Between baseline and follow-up, anti-tetanus vaccination rates increased from 69.0% to 85.0%, and early breastfeeding also increased, from 42.9% to 57.5%. More newborns were checked by trained health workers (39.2% to 75.5%), and women were performing more of the critical newborn care activities at follow-up. Fewer women relied on the traditional birth attendant for health advice (48.4% to 11.0%, with corresponding increases in advice from trained health workers. At follow-up, most of these improvements were greater in the intervention than control communities. In the intervention communities, there was less use of anti-malarials for all symptoms, coupled with more use of other medications and traditional, herbal remedies. Infant and child mortality declined in both intervention and control communities, with the greatest declines in intervention communities. In the intervention communities, infant mortality rate declined from 90 at baseline to 59 at follow-up, while child mortality declined from 160 to 84.

**Conclusions:**

These results provide evidence that in the context of ongoing improvements to the primary health care system, the participatory and community-based interventions focusing on improved newborn and infant care were effective at changing infant care practices and outcomes in the intervention communities.

## Background

In 2011, two-thirds of the 7 million deaths of children under the age of five could have been prevented by low-cost, integrated newborn and child heath (NCH) interventions [1,2]. The majority of global childhood deaths occur at home, without any contact with the formal health system [3]. It has therefore been suggested that interventions will be effective only if they reach women and children in the home [4], and can include basic preventive and curative activities that women can do at home: early initiation of breastfeeding, early postnatal follow-up care of newborns, exclusive breastfeeding for at least six months, recognition of danger signs of illness, and case management of acute illnesses during early childhood [2,3,5,6]. As integrated maternal, newborn, and child health (MNCH) packages are now being delivered to-scale across many low-income countries, there has been an acceleration in the decline of global childhood mortality since 2000.

However, in Sub-Saharan Africa, reductions in childhood mortality have been slower than in the rest of the world [1]. While there are a number of contributory factors to this regional disparity, a major obstacle is the difficulty women have in obtaining competent and appropriate health worker care [7]. Countries with higher density of health professionals per capita have higher rates of infant and child survival, yet Sub-Saharan Africa has the lowest health worker density in the world at 2.3 per 1,000 population [8]; in rural areas the density is actually much lower than this since health workers are concentrated in the urban regions [9]. In light of these obstacles to care, community health workers (CHWs) have emerged as a solution with the strongest potential to strengthen primary healthcare delivery in sub-Saharan Africa [10,11]. CHWs are described as “members of the communities where they work, should be selected by the communities, should be answerable to the communities for their activities, should be supported by the health system but not necessarily a part of its organization, and have shorter training than professional workers” [12]. The effective use of CHWs has the capacity to address the three major gaps in service delivery: coverage, equity, and quality [7]. Further, emerging evidence suggests that this cadre of health worker is uniquely capable of reaching the most vulnerable, from the poorest families and living in remote areas of Sub-Saharan Africa [4,13-21].

In Nigeria, the most populous country in Africa and a country with the second highest burden of child deaths in the world, the need to improve child survival is paramount [3]. Further, within Nigeria there are marked differentials in child mortality rates, with rates in the northern states two to three times higher than in the southern states [14]. In response to this need, the Partnership for Reviving Routine Immunization in Northern Nigeria (PRRINN) was established in 2006 in four northern states of Nigeria (Jigawa, Katsina, Yobe, and Zamfara) and then, in 2008, expanded to include maternal, newborn, and child health (PRRINN-MNCH). The program is comprehensive, encompassing multiple aspects of the health system including human resources, health governance, health information, strengthening of clinical services and community engagement in order to reduce maternal, newborn, and child mortality. The strategy adopted is to focus on revitalizing comprehensive primary care services using a cluster approach which builds capacity for the provision of emergency obstetrical care services at selected facilities, with strong primary care facilities support care and referrals to these designated centers in each cluster. Attention is paid to training of health care workers at all levels in this cluster, as well as strengthening demand for health care services within communities served by these designated emergency care facilities. The program utilizes an operations research approach that promotes evidence-based quality improvement of ongoing program activities.

A key element of this integrated strategy is the development of a network of CHWs, who bridge between the household and the health facility. To date, there has been relatively little attempt to incorporate community health workers into community-based promotion of primary care in Nigeria, although there is evidence that they would be effective [22]. Nigeria has adopted the UNICEF/WHO program for the Integrated Management of Newborn, Infant, and Childhood Health (IMCI), but to date the implementation of IMCI has been limited to selected Local Government Areas, and has generally not included the community IMCI [23]. The PRRINN-MNCH program therefore developed a two-pronged approach to the implementation of community IMCI: Community volunteers, who translate the IMCI prevention messages into participatory dialogues, consistent with a pilot in southern Nigeria, [23] and trained community health extension workers (CHW), delivering the diagnosis, treatment and referral elements of C-IMCI, modelled after the Ethiopian experience [20].

This combination of participatory group discussions with a CHW has been piloted in Nepal and other parts of Asia and Africa, but seldom in the context of a functional primary health care system, and mixed results regarding the combined effect of participatory women’s group and CHW may relate to the low level of functionality of the health care system [24-27]. The integrated nature of the PRRINN-MNCH Program offers a unique opportunity to assess the impact of the community interventions in the context of carefully integrated elements throughout the primary health care system. Specifically, we will examine the extent to which this integrated approach in these states has facilitated improvements in the key behaviors and outcomes of the IMCI program, namely, newborn and child health care knowledge and behaviors among caregivers, and changes in newborn and child morbidity and mortality.

## Methods

### Study site

The study was conducted in the three northern Nigerian states where PRRINN has expanded its MNCH activities, namely Katsina, Yobe, and Zamfara, with respective populations of 5.8, 2.3, and 3.3 million, according to the 2006 population census of Nigeria. These states are part of the Sahelian zone, with an alternation of dry and wet seasons. The program focuses on the rural communities, where most of the residents are subsistence farmers, pastoralists, or traders. The level of literacy is low, particularly among women, where over 80% are unable to read or write. Half or more of the women do not listen to the radio or watch television at least once a week. Compared to the southern zones of Nigeria where primary care use is widespread, there is very little primary care service use in the northern zones of Nigeria. In these states, less than 50% of women had any antenatal care in their last pregnancy, over 85% delivered at home without assistance of a skilled attendant, and about 75% had no postnatal care. These states had the lowest immunization coverage rates, under 10% of 12–23 month olds, and the lowest rates of utilization of health care services for treating sick children.

### Intervention design

The intervention design reflects the integration of two different approaches to the improvement of health care. First, the design uses the PRECEDE-PROCEDE framework for health promotion through system-wide changes in health planning and implementation which facilitate changes in MNCH health knowledge, practices, and outcomes, incorporating participatory methods and operations research to assess progress in achieving changes at each stage [28]. Second, the intervention design is implemented spatially through a cluster approach, which focuses on reducing the three delays to emergency obstetrical care [29-31]. The program focuses on improving MNCH care by clusters of Local Government Areas (LGAs) per state, which each comprise a catchment area for emergency obstetrical care (EOC) services, with one Comprehensive EOC facility per 500,000 people. Making referral to this Comprehensive EOC facility are four Basic EOC facilities (each serving 100,000 people with the Comprehensive EOC facility serving the other 100,000 people) and eight “24/7” facilities providing maternal care. A total of 15 LGAs were selected as the first intervention clusters, 4–6 per state. The remaining LGAs had statewide policy changes without focused clinical or community activities to improve health system infrastructure and MNCH care demand, and served as the control communities.

In addition to the development of EOC capabilities, the health system strengthening component of the intervention included midwife training and posting through the Nigerian government’s Midwife Service Scheme, establishment of planning and management techniques within existing facilities, strengthening distribution of essential drugs to PHC facilities, refurbishment of PHC equipment (as needed), training in IMCI for the PHC clinical staff, and establishing the “Primary Health Care Under One Roof,” which consolidates and coordinates the different components of primary care in one health clinic or post.

Complementing these supply-side changes, are activities that create demand for MNCH services. Selected groups of villages served by primary care facilities linked to the upgraded EOC facility participate in a community engagement process, which aims to increase awareness, knowledge and practices of healthy behaviors in response to MNCH barriers. Core to this process is a participatory, community discussion group, facilitated by trained community volunteers (CVs).

The community volunteers have been recruited and trained in each community, using a cascade or train the trainers model, with core trainers training CVs who in turn train new volunteers. The participatory training methodology is underpinned by key principles of adult learning, starting with discussions and reflection on personal experiences, which in turn are used to discuss potential responses/solutions women could adopt. Body memory tools (e.g., mimicking movements of the body when affected by different danger signs) help trainees remember key facts. By 2011 CVs, primarily women, had been recruited, mobilized and supported in their work in all the intervention communities. The primary responsibility of the CV is to facilitate community discussion groups through the use of jingles and other visual-auditory cues to educate about critical MNCH issues, such as danger signs for a pregnancy. In addition, the CVs also aid in identifying at risk women and children and referring them to the nearest facility.

These dialogues provide an opportunity for reflection and problem solving on the most prevalent MNCH problems affecting the community. Members of the discussion groups are encouraged to share what they know with their families and peers between sessions. Community discussion group participants are encouraged to put what they have learned into practice by tackling key barriers of access to and affordability of MNCH services, including establishment of blood donor groups, community emergency savings schemes, community emergency transport schemes and a “mother’s helpers” system. This work is reinforced by mass communication activities, including the use of radio “jingles” to promote birth preparedness or childhood immunizations.

The CV have been complemented by a small cadre of CHWs, community-based health workers providing selected primary health services directly to families through rotating visits or extended availability through residence in the communities, similar to the models used in other countries with volunteers and paid CHWs. These CHWs were recruited among unemployed Junior Community Health Extension Workers, previously trained by the state Schools of Health Technology, who were then given two weeks of additional training and toolkits to enable them to make home visits, engage mothers using supportive communication techniques, provide basic preventive antenatal, newborn, and child care, basic treatment and referral to the PHC, according to the Integrated Management of Newborn and Childhood Illnesses protocol. These CHW-Community Based Service Delivery (CBSD) are provided with transport to enable them to visit communities on a regular schedule, and they spend most of their time visiting families in the community.

### Evaluation design

The assessment of the impact of the CBSD programs uses a quasi-experimental design using pre- and post-intervention household surveys in the intervention and control communities. The pre-intervention or baseline household survey (BHS) was conducted in 2009 and the post-intervention household survey, the follow-up household survey (FHS) was conducted in 2011. The evaluation of the impact of this integrated MNCH package takes into account both availability of program and actual individual participation in any of the program’s community-based service activities. Availability of the program activities was assessed by comparison of intervention and control areas. Individual exposure to the program was assessed by the woman’s responses to questions eliciting sources of information or health care advice. The study was approved by State Ethics Review Committees in each of the three states, as both a cross-state and individual state approval. These ethics review committees are certified by the Nigerian Federal Government’s National Health Research Ethics Committee to review and approve health research protocols for their states.

### Study sample

The sampling plan was a stratified two-stage cluster sample, with oversampling of individuals in the MNCH intervention clusters. Individuals from MNCH clusters were oversampled using a ratio of 2:1, because MNCH clusters cover a significantly lower proportion of the population of each state. Oversampling therefore provided a sufficient sample in the intervention areas to assess the impact of key elements within the intervention package on the key MNCH outcomes. The PSU for this sample was the Local Government Area (LGA), for which there were 24 in the BHS and 15 in the FHS. For the FHS, the same intervention LGAs as the BHS were included, with the exception of LGAs of the state capitals (considered not an appropriate control for the largely rural intervention). The LGAs comprising the state capitals were included only during the baseline to assess the differences in services provided to residents patronizing urban versus rural facilities. This enabled the team to devise appropriate strategies for referral from rural to urban facilities. The state capitals were excluded in the analyses reported here. The number of households selected per LGA was proportional to the size of the LGA.

The study was designed with an 80% power to detect a 2.5% change in the percentage of women delivering with the assistance of skilled birth attendants between the BHS (11%) and the FHS. The BHS was designed to be representative of all ever-married women in the household and required a sample of 5,560 households, while the FHS was designed to be representative only of ever-married women with a birth in the previous 5 years, requiring a sample of only 2,310 households. In the BHS, the sample of 5,560 households was 0.7% to 9.8% per LGA, while for the FHS the 2,310 households comprised 3.1% to 13.1% of all households.

Within the LGA, the sample of households was allocated to intervention and control communities in proportion to the size of the community or village. The sampling fraction for each community was determined by information on the total households from the community leadership. Households within each selected community were randomly sampled using a procedure similar to that used in the WHO-EPI cluster surveys, namely by numbering then sampling households according to the community sampling fraction along randomly selected paths leading out from the center of the village.

The household was the ultimate sampling unit. In compounds that comprised one to three households, one household was randomly chosen for interviews; in compounds with four to six households, two were surveyed; in compounds with seven or more households, three were surveyed. Within each randomly selected household, in the baseline survey, all ever-married women of childbearing age (15–49 years) were interviewed, whereas in the FHS only one ever-married women with at least one child born in the last 5 years was selected for interview. The inclusion criteria were changed for the follow-up survey because of the need to focus on women with pregnancies and births during the time period during which the intervention was implemented. In the BHS there were 6,842 women with successfully completed interviews, while in the FHS there were 2,310 completed interviews.

Interviewers who had completed secondary school or higher were selected and trained to visit the selected women at home and administered a questionnaire that included translation of key concepts and terms in the local languages (e.g., Hausa, Kanuri). Most of the interviewers were females, responding to cultural expectations and beliefs that encourage female interviewers to interview female respondents. In both the BHS and FHS, the questionnaires used adopted some of the close-ended questions from the 2008 Demographic and Health Survey [14] to allow comparisons of results with other national- or state-level data. Questions were modified in line with the program goals and focused on a series of topics related to perceptions, knowledge, and practices related to MNCH outcomes. Specifically, the topics included issues related to information such as age, parity, economic status, literacy in any language, wife rank, antenatal care and delivery characteristics, source of health advice for the woman or the baby during last pregnancy, experience of labor and delivery complications, knowledge of maternal and newborn danger signs and how to respond to them, actual response to danger signs of infant and child illness, and infant and child mortality.

### Analysis

At the analysis stage, the inclusion criteria for both surveys was narrowed to ever-married women, aged 15–49, with a birth in the previous five years. Respondents were assigned to the control or intervention groups based on the level of PRRINN-MNCH program intervention at the time of the survey. For the BHS, which was pre-intervention, the intervention LGAs included all LGAs in the first cluster receiving CEOC upgrades and related community engagement activities, while the balance were control LGAs. The FHS included the same intervention LGAs, but control LGAs in 2009 were shifted into the intervention category if they had started to receive the primary health care and community health worker interventions by the time of the FHS. The dependent variables are the key health behaviors pertaining to maternal, newborn care and care of sick children. Infant and child mortality rates were calculated using standard demographic estimation methods. The infant and child mortality rates were calculated using the retrospective reports of births and deaths in the previous 12 months and five years, per the standardized format of the Demographic and Health Surveys. Rates were calculated separately for each survey period, aggregating the reported births and deaths per household. We first verified the number of births and deaths for the appropriate reference period (one or five years) using the built-in cross-referencing between questions, excluding implausible values (e.g., deaths to children under five exceeding births, after controlling for children moving in and out of the household), and then calculated the mortality rates using the appropriate births denominator. The bi-variate and multi-variate analyses of the two sets of survey data were conducted separately, each using sampling weights based on the intervention and control areas. We examined changes in the proportion with the designated MNCH behaviour or outcome, contrasting all pre-intervention responses (all BHS) versus the post-intervention responses from the FHS, intervention versus control. We assessed the degree to which the intervention and control groups differed using the Chi-square statistic. Analyses were performed using Stata 12.0 (Statacorp, College Station, TX) and SPSS version 19.0 (SPSS Inc. Chicago, Ill).

## Results

### Respondent characteristics

After including only ever married women with births in the previous five years, there were fairly small differences between the two groups (See Table 1). In both rounds, most women interviewed were between the ages of 20 and 34 years, with the greatest concentration in the 25–20 age group. Virtually all women interviewed were currently married, and about 80% were monogamously married. Over 80% of women had no formal schooling, and among those with some schooling, and there were no significant differences in the level of schooling among those who had attended school. The majority of women in both surveys could not read or write Hausa, the language of these states. Significantly more women described themselves as housewives in the follow-up survey than at baseline (43.6% versus 34.6%, t = 12.7), with corresponding declines in the proportions engaged in food processing and farming. Those in trading/selling increased from 18.1% to 26.4%. Women interviewed in the FHS also were more likely to have access to a cell phone (31.7% vs. 7.5%, t = 31.6). At follow-up considerably more were likely to be in communication with women elsewhere, through access to a cell phone.

**Table 1:** Background characteristics of respondents, Northern Nigeria, 2009 vs. 2011

**Background characteristics**	**BHS 2009**	**FHS 2011**
	**Number (%)**	**Number (%)**
*Age group (years)*		
15–19	261 (12.3)	179 (7.8)
20–24	460 (21.6)	529 (22.9)
25–29	548 (25.8)	608 (26.3)
30–34	373 (18.4)	526 (22.8)
35–39	234 (11.1)	281 (12.2)
40–44	134 (6.3)	147 (6.4)
45–49	100 (4.7)	29 (1.3)
*Marital status*		
Married	2072 (97.7)	1581 (99.0)
Widowed	23 (1.1)	5 (0.3)
Divorced or separated	24 (1.1)	10 (0.6)
*Rank of wife*		
1	1657 (79.6)	1182 (76.2)
2	368 (17.7)	301 (19.4)
3 +	54 (2.6)	68 (4.4)
*Formal education*		
Yes	343 (16.2)	219 (13.9)
No	1772 (83.8)	1358 (86.1)
*Level of formal education (if any)*		
Primary	236 (66.1)	155 (72.1)
Secondary	53 (14.9)	34 (15.8)
Post-secondary	68 19.0)	26 (12.1)
*Reading and writing in Hausa*		
Not at all	1785 (84.2)	1392 (89.5)
With difficulty	142 (6.7)	82 (5.3)
Easily	193 (9.1)	81 (5.2)
*Occupation*		
Food processing	647 (30.5)	449 (28.5)
Agricultural processing	100 (4.7)	70 (4.5)
Farming	100 (4.7)	0 (0.0)
Trading/Selling	384 (18.1)	410 (26.4)
Housewife	734 (34.6)	682 (43.6)
Other	157 (7.4)	105 (6.7)
*Cell phone access*		
Yes	160 (7.5)	500 (31.7)
No	1966 (92.3)	1077 (68.3)
**Number**	**2,129**	**2,310**

*Notes:* Some percentages may not add up to 100 due to rounding of decimals. Some numbers for sub-categories may not add up to the total due to missing values.

All differences in proportions of main categories are not significantly different (t-test) at .05 level, except the proportions who are housewives (t = 12.7) and cell phone access (t = 31. 6).

The majority of the households (about 80% both surveys) included only one family, but if the household had more than one family in the compound, there was an average of 2.5 families living together. There are an average of four women living in each household, and of these women, on average 1.5 had given birth in the past year.

### Newborn and child health outcomes

**Figure 1:**
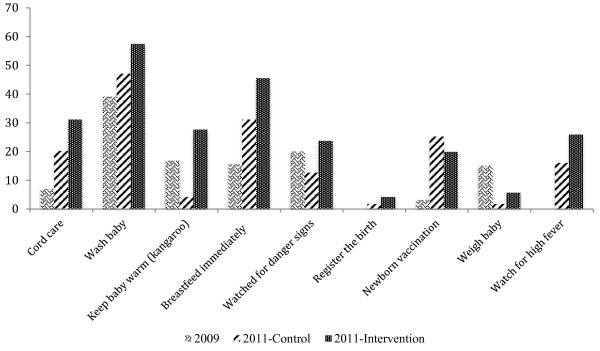
Percent of newborn care activities by intervention area, Northern Nigeria 2009 and 2011.

In 2011, more infants were protected from tetanus. The proportion of women who had received anti-tetanus vaccinations had increased from 69.0% to 85.0%, with the increases equal in the control and the intervention areas. There was a significant increase in the proportion newborns first breastfed within 24 hours from birth, from 42.9% to 57.5%, with significantly more (60.5%) in the intervention areas (Table 2). Fewer infants had a postnatal check by a health worker within 48 hours of birth, down from 39.2% at baseline to 27.5% in the intervention and 18.9% in the control areas. However, there was a large change in who checked on the newborn. At baseline, the majority of newborns were checked at home by the traditional birth attendants (TBAs) (40.8%), while at the mid-term most newborns were checked by a nurse/midwife at the health facility, 51.3%% in the control areas and 38.6%% in the intervention areas. More newborns were checked by CHWs, with even more in the intervention (46.3%) than control areas (35.8%). There was a significant increase in newborn care provided to the infant: cord care from 7.0% to 26.4%, washing the baby in warm water from 39.1% to 52.9%, kangaroo care from 16.8% to 17.5%, breastfeeding within eight hours from 15.6% to 39.3%, and newborn vaccinations from 3.1% to 22.2% (Figure 1). Consistently, the provision of newborn care elements was significantly greater in the intervention than in the control zone, the sole exception being newborn vaccinations. At the midterm in 2011, significantly more women were told about how to care for their newborn, up from 68.1% to 71.6%, with even more informed in the intervention than control areas. The other major change was a shift from relying on the TBA for information about newborn care (from 48.4% to 11.0%) to CHWs (from 6.8% to 11.7%, and further increase to 13.9% in the intervention communities). The impact of the community discussion groups was seen in the large share of women learning about newborn care from women’s groups, friends, and family.

**Table 2:** Newborn care for the most recent birth, by intervention area, Northern Nigeria, 2009 vs. 2011

**Characteristic**	**BHS 2009 (%)**	**FHS 2011 (%)**	**Control 2011 (%)**	**Intervention 2011 (%)**	**p-value BHS vs Int**	**p-value Ctl vs Int**
Mother had anti-tetanus vaccine	69.2	85.0	84.8	85.1	<0.0001	0.9170
n	1,335	976	244	732		
First breastfeeding within 24 hours	42.9	57.5	54.1	60.5	<0.0001	<0.0001
n	1,335	2,305	729	1576		
First postnatal check within 48 hour	39.2	24.1	18.9	27.5	<0.0001	0.0120
n	1,335	1,753	589	1,164		
*Person checking newborn*						
Nurse/midwife	34.5	44.1	51.3	38.6	<0.0001	<0.0001
CHW- health post	4.7	30.8	27.3	34.1	<0.0001	0.0160
CHW- outreach	NA	10.6	8.5	12.2	NA	0.0730
TBA	40.8	2.8	2.6	2.9	<0.0001	0.8500
Other	20.0	11.8	11.1	12.2	<0.0001	0.6070
n	1,335	679	189	490		
*Care provided to the newborn*						
No special newborn care	NA	1.1	0.83	1.3	NA	0.0040
Cord care	7.0	26.4	20.2	31.1	<0.0001	0.0040
Wash baby	39.1	52.9	47.1	57.4	<0.0001	0.0160
Keep baby warm (kangaroo)	16.8	17.5	4.2	27.6	<0.0001	<0.0001
Breastfeed immediately	15.6	39.3	31.1	45.5	<0.0001	0.0010
Watched for danger signs	20.0	18.9	12.6	23.7	0.9147	0.0010
Register the birth	NA	3.1	1.7	4.2	NA	0.0950
Newborn vaccination	3.1	22.2	25.2	19.9	<0.0001	0.1350
Weigh baby	15.1	4.0	1.7	5.7	<0.0001	0.0180
Watch for high fever	NA	21.6	16.0	25.9	NA	0.0050
n	1,441	2,305	729	1,576		
Source of information about newborn care*						
No one	31.9	28.4	34.0	23.3	<0.0001	<0.0001
Nurse/midwife	25.0	6.4	6.2	6.5	<0.0001	0.6830
CHW in health post	6.8	7.7	6.0	9.3	<0.0001	0.0010
CHW in outreach	NA	4.0	1.9	4.6	NA	<0.0001
TBA	48.4	11.0	16.7	5.6	<0.0001	<0.0001
Family/ friends	NA	27.4	30.7	25.3	NA	0.0010
Drug vendor/ Chemist	NA	0.3	0.5	0.0	NA	0.0030
Other	1.8	3.0	2.5	3.4	<0.0001	0.1450
**Number of women**	**2,129**	**2,305**	**729**	**1,576**		

Notes:^*^Source of information about newborn care is the person checking the newborn and counseling the mother after delivery; “CHW” – Community Health Worker; “TBA” – Traditional Birth Attendant; “NA” – Not applicable; “Ctl” – Control area’ “Int” – Intervention area; “BHS” – Baseline Household Survey; “FHS” – Follow-up Household Survey.

At the midterm follow-up, most women knew at least one of the newborn danger signs, with the most commonly known danger sign being high fever, known by 82.7% in the control and 84.2% in the intervention communities (Table 3). Many women knew other critical danger signs that indicated the need for the baby to be seen by a health worker. In the intervention areas, 31.0% knew to worry about diarrhea, dehydration, and sunken fontanel and about fitting or convulsions, significantly more than in the control areas. Women in the intervention areas were also more likely to know about breathing problems and not being able to suckle or refusing to feed. About half (47.2%) of the newborns experienced at least one of these danger signs during the first six weeks of life, with no difference in the frequency between control and intervention areas. Of those with one of the danger signs, the most common danger sign was fever, with significantly more of the newborns having fever in the control (31.8%) than intervention communities (27.8%). One in five newborns in the intervention communities (20.6%) cried excessively, noted by significantly more mothers in the intervention than control communities (16.6%). More newborns in the intervention versus control communities were also noted to have breathing problems (18.0% vs. 14.9%). The next most common danger signs were diarrhea and swollen stomach, each experienced by about 16% of all newborns in the control areas and 19% in the intervention areas.

**Table 3:** Knowledge of and response to newborn danger signs and illness episodes of children under age 5 years by intervention area, Northern Nigeria, 2011

**Newborn danger sign knowledge and response type**	**Control 2011 (%)**	**Intervention 2011 (%)**	**p-value (Control vs. Intervention)**
*Knowledge of newborn danger signs*			
None Known	12.3	9.2	0.0200
Known:			
High fever	82.7	84.2	0.3830
Stiff neck, fitting or convulsions	16.4	31.2	<0.0001
Jaundice	3.5	18.4	<0.0001
Difficult/fast breathing	14.2	20.6	<0.0001
Not able to suckle/refuse to feed	8.3	15.6	<0.0001
Diarrhea/dehydration/sunken soft spot	20.1	30.5	<0.0001
n	422	1,100	
*Observation of danger signs in newborn (<6 weeks old)*			
High fever	31.8	27.9	0.0170
Stiff neck, fitting or convulsions	6.6	5.3	0.1440
Swollen stomach	15.5	18.1	0.0580
Diarrhea	16.7	19.5	0.0450
Difficult/fast breathing	4.4	7.4	<0.0001
Not able to suckle / refuse to feed	5.8	6.5	0.3760
n	729	1,576	
*Source of advice on care of sick children*			
Nurse/midwife	11.4	12.1	0.9390
CHEW in health post	10.7	18.2	<0.0001
CHEW in outreach	3.8	5.6	0.6400
TBA	7.7	1.9	0.1920
Family/ friends	32.2	27.1	<0.0001
Drug vendor/ chemist	2.6	1.0	0.0130
Traditional healer/ other	0.6	0.4	0.0460
No one mentioned	28.3	22.5	0.3010
n	248	579	
*Acute illness episodes in past month, children <5 years*			
Fever	24.4	28.4	0.0370
Diarrhea	15.4	19.4	0.0150
Cough	11.4	15.2	0.0080
Malnutrition (weight loss)	11.1	15.2	<0.0001
Fever and cough	9.0	13.2	<0.0001
Diarrhea and malnutrition	8.3	11.8	0.0020
n	729	1,576	

One-third (32.6%) of the households reported a sick child (under age 5 years) in the month prior to the interview. The average duration of illness was 7.8 days. The most common illnesses were fever (presumed to be malaria) (26.5%), diarrhea (17.4%), cough (13.3%), and malnutrition or weight loss (13.7%). One in ten (11.2%) children had both fever and cough, as did another 10.1% who had diarrhea and malnutrition (see Table 3). The reported illness prevalence rates were slightly higher in the intervention than control communities.

Between the BSH and FHS in 2011, Table 2 shows there was a shift in the source of advice about the care of sick children. More women in the intervention communities knew about the care of their sick children, with only 22.5% in the intervention areas and 28.3% in the control areas having no one to teach them about the care of their sick children. More women learned how to care for sick children from CHWs, both at the health post and in the community, with CHWs providing this information to 14.5% in the control communities and 23.8% in the intervention communities. Fewer relied on family and friends in the intervention communities, 27.1% vs. 32.2% in the control communities. In the intervention communities, fewer women went to a TBA or drug vendor/ chemist for advice on treating a sick child.

In both time periods and regardless of the child’s symptoms, about one-third of all mothers with sick children in the past month reported seeking no advice and providing no special care to the sick child (see bottom row, Table 4). Approximately, one in twelve (8%) mothers in the intervention communities gave additional fluids, including breastfeeding more, to their children with fever, cough, fever and cough, and diarrhea, compared to slightly fewer giving fluids in the control communities. Although the reported rate of giving ORS declined between the BHS and FHS, in the intervention communities over one in ten (9.2% to 13.8%) gave oral rehydration solution (ORS), compared to an ORS usage rate of 5.0% or below in the control communities.

**Table 4:** Type of care given to sick child in the month preceding the survey by intervention area, Northern Nigeria, 2009 and 2011

**Type of care (%) (n with any care)**	**2009 Fever/cough**	**2011-Fever only**		**2011-Cough only**		**2011-Fever and cough**		**2009-Diarrhea**	**2011- Diarrhea**	
	**n = 1,205**	**n = 625**		**n = 323**		**n = 274**		**n = 1,335**	**n = 417**	
***Control vs. Intervention***		***C***	***I***	***C***	***I***	***C***	***I***		***C***	***I***
**Homecare**										
Gave more fluids	NA	7.9	7.2	6.0	8.3	7.6	8.2	NA	**5.4**	**13.8**
Gave ORS	**18.9**	**3.9**	**9.2**	**1.2**	**9.2**	**1.6**	**10.1**	**32.7**	4.5	7.5
**Medication use**										
Analgesics	39.0	37.6	36.3	32.1	32.5	35.6	32.7	29.9	33.8	32.7
Antibiotics	35.9	26.4	32.2	37.7	31.4	31.1	34.0	36.2	37.7	34.6
Anti-malarial	**57.5**	19.2	18.2	20.8	20.1	24.4	20.4	**55.8**	**10.4**	**18.2**
Gave cough medicine	NA	11.2	13.4	28.3	23.1	26.7	23.8	NA	11.7	12.6
**Did nothing**	35.2	29.8	29.8	36.1	29.7	31.8	29.5	**40.0**	31.2	**29.8**
**Total Sick**	**2,910**	**178**	**447**	**83**	**240**	**66**	**208**	**1,415**	**112**	**305**

*Notes:***Bold** indicates a p-value of <0.05 between intervention and control; Percentages do not sum to 100% because multiple care activities may have been used per episode; “C” and “I” refer to control and intervention areas, respectively.

Over one-third used an analgesic (paracetamol) to reduce fever at both baseline and mid-term follow-up, with more using analgesics for diarrhea at mid-term than at baseline (Table 4). Approximately, one-third of all mothers also used antibiotics to treat their children’s fever, cough, or diarrhea. Use of antibiotics generally was less at the mid-term than baseline, but there were different directions of change in the control and intervention communities. Antibiotic treatment of fever dropped to 26.4% in the control communities, compared to 32.2% in the intervention communities. A similar pattern was seen for the treatment of fever and cough. The reverse was seen for cough, for which only 31.4% of the mothers in the intervention communities gave antibiotics, compared to 37.7% in the control communities. This alternate pattern was also seen for antibiotic treatment of diarrhea, higher in the control (37.7%) than intervention (34.6%) communities. Use of anti-malarials dropped precipitously between the baseline, when 57.5% used them for fever and/or cough, down to 20% or less in both the intervention and control communities at the follow-up. About a quarter of mothers in both the control and intervention communities also reported using cough medicine for cough, with or without fever.

**Figure 2:**
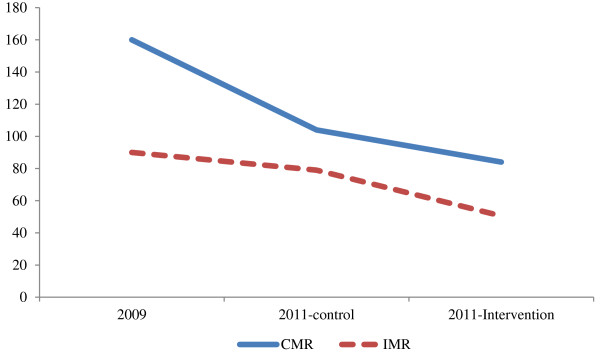
Infant and child mortality rates by intervention area, Northern Nigeria, 2009 versus 2011.

Between 2009 and 2011, there was a decline in both the infant and the under-five mortality rates (see Figure 2). In 2009, the infant mortality rate (IMR) was 90.0 deaths per 1,000 live births, and this fell to 79.0 in the control communities and 50.5 in the intervention communities, averaging 58.5 in both. During this same period, the child mortality rate declined from 160 to 104 deaths per 1,000 children in the control communities and 84 in the intervention communities.

## Discussion

Although the PRRINN-MNCH Programme only had been underway for just over two years when the FHS was conducted, there already was evidence of significant improvement in several of the key newborn, and child health behaviors and outcomes. The level of newborn and child morbidity remains high in these communities, with almost half of all newborns reported to show one of the danger signs and one-third of young children experiencing an illness episode in the previous month. Nonetheless, we observed a significant decline in infant and child mortality at follow-up. Further, the mortality decline was greater in the intervention than the control areas. This is a remarkably fast period of time to observe a mortality decline, and particularly when there was not a huge change in the proportions delivering in a health facility where newborn care would be included as part of the delivery. We believe that the change in infant mortality may result from how women respond to their infant’s health when they realize that health is threatened. Compared to the baseline in 2009 when women were less likely to seek advice and respond as quickly to infant danger signs or advice about newborn care, at follow-up in 2011 women in the intervention communities were very much alert to and responding to these dangers.

Fairly consistently, the intervention communities displayed improved neonatal and newborn care practices. More women in the intervention than control communities started breastfeeding immediately and exclusively, had their newborn checked by a health worker, washed their baby in warm water, kept the infant warm with kangaroo care, and knew to watch the infant for fever or other danger signs. These all were topics addressed in the community discussion groups organized by community volunteers recruited and trained by the project, and the changes in care of the newborn immediately after delivery reflect this increased community dialogue, awareness and support for the need to keep an eye on the mother and newborn during this critical period. Our findings of the increased knowledge about danger signs and improved care for newborns is consistent with the findings of other programs which include community volunteers leading participatory dialogue groups [24,27,32,33].

Our study, however, also documents changes in interactions with staff of the primary health care facility. The community dialogue encouraged women to go to the community health worker or the health worker at the closest health post instead of the TBA, because these trained individuals can do more to help the mother recognize and respond to any problems that might develop. The follow-up survey documented the increased reliance on community health workers care of the newborn or sick child. At baseline, almost half of all newborns were checked by a TBA, whose activities generally were limited to assistance with cord care and cleaning up the baby. At the follow-up two years later, hardly any women took their newborn to be seen by a TBA. Instead, they had their babies checked by the midwife or CHW at the health post or by a CHW making an outreach visit to the community. A similar pattern is seen for seeking advice on the care of sick children, with the intervention group showing increased reliance on the midwives and CHWs and less on the TBA or family and friends. At follow-up, women in the intervention communities also were more likely to have received anti-tetanus vaccinations at the health post. We believe that the simultaneous improvement in the quality of care provided by the CHW and nurse-midwives at the health post gave women the confidence that they could go to the health post to seek advice and care. Thus, our study shows that when the participatory community dialogues are accompanied by supportive primary health care structures, including community health workers reaching out to families and improved quality of service in the clinics, women are more likely to adopt both the clinical and at-home newborn care recommendations, as suggested in earlier reviews of newborn care interventions [26,33].

Another critical change in newborn care is the increased understanding and ability to observe newborn danger signs. In the intervention communities, more women knew newborn danger signs and they also knew more of them. Mothers in the intervention communities reported higher incidence of some of the newborn danger signs, but this likely reflects their new found understanding that these signs are not “normal” and require the mother to have her child treated quickly. Women reported that almost half of their newborns had shown at least one of these danger signs, with the most common being a high fever. It is likely that this level of observation was connected to taking steps to obtain urgent care, as that is what the danger sign message is all about. The higher quality and availability of care at the primary health centers would have given women confidence that arriving their with their newborn could indeed be a life-saving trip.

At follow-up, mothers also were responding with more home care for their sick children. When their children had fever and diarrhea, at follow-up one in ten women in the intervention communities reported treatment with home mixed sugar-salt solution, as instructed by the CHW. In the control communities, there was hardly any use of ORS in 2011. The mothers in the intervention communities also reported giving more fluids to their children, though the differences were not significant between the control and intervention areas. Both changes reflect the influence of the community discussions in the intervention areas, where women in the community learned about the importance of rehydration and then through CHW home visits how to mix the sugar-salt solutions. Our results suggest that the combination of community dialogues plus CHW home visits can address one of the priority problems identified by the Child Health and Nutrition Research Initiative, namely to identify methods to increase the practice of home care to prevent and treat newborn infections [34].

This study has several limitations. First, we did not combine the datasets for an integrated analysis of the behavior change between baseline and midterm, which limits the analysis to a comparison of means and proportions. The pre-post comparison between the BHS and the End-of-Project Survey will include this merger of data, enabling regression analyses to be used to identify the predictors of behavior change and health outcomes. Second, all behaviors and health outcomes are by self-report, with no medical verification of the health events or deaths. As in any retrospective self-report, particularly of infant and child deaths, there is likely to be under-reporting. We assume that the level of under-reporting for these events is comparable across both surveys, but with the increased push for birth registration by the programme, it is possible that the reporting of births and deaths is higher in the FHS, which would tend to upwardly bias the mortality estimates relative to the BHS. Third, and most importantly, the CHW intervention components were only operational for approximately one year prior to the FHS, and hence the period of exposure is more limited for these elements of the intervention. The lack of change for some indicators between the BHS and FHS may therefore be due to limited exposure to the intervention. The End-of-Project Survey will permit a longer duration of exposure to be assessed, and it will include a more detailed set of measures of program participation. These limitations notwithstanding, the net result of the changes in understanding about newborn and sick child care were evident in the observed declines in infant and child mortality rates during this short time period. Declines in both rates were observed in both the control and intervention areas, but they were significantly greater in the intervention communities.

## Conclusions

Prior to launching the PRRINN-MNCH program, infant and child mortality rates were very high; there was very little use of the primary health care system, and virtually no community-based services. The program used an integrated approach which simultaneously matched quality improvements in the primary health care system with community-based health promotion activities which built confidence and communitysupport for women learning about and taking care of their children, including through use of primary health care services. This comparison of newborn and infant care practices in the intervention and control communities provides evidence that in the context of ongoing improvements to the primary health care system, the participatory and community-based interventions focusing on improved newborn and infant care were effective at changing infant care practices and outcomes in the intervention communities.

## Competing interests

Authors declare that there are no competing interests.

## Authors’ contributions

SF, OU, HD, CG, FA, and GYA participated in the conception of the study, assisted with the analysis, interpretation, drafting, and revision of the manuscript. All authors read and approved the final manuscript.

## Pre-publication history

The pre-publication history for this paper can be accessed here:

http://www.biomedcentral.com/1471-2458/13/1034/prepub
